# Building capacity and advancing collaboration in dissemination and implementation science: insights from a multi-institutional partner network

**DOI:** 10.3389/frhs.2026.1877155

**Published:** 2026-09-07

**Authors:** Jing Li, Naomi Duffort, Ehsan Abdalla, Khalilah Brown, Sara Cooper, Sara L. Davis, Thomas Dobbs, Kirsten Dorans, Karen Johnson, Kristine Ria Hearld, Peter T. Katzmarzyk, Benjamin Springgate, Renee Heffron, Salisa Westrick, Michael Mugavero

**Affiliations:** 1 Department of Medicine, The University of Alabama at Birmingham, Birmingham, AL, United States; 2 Johns Hopkins University, Baltimore, MD, United States; 3 College of Veterinary Medicine, Tuskegee University, Tuskegee, AL, United States; 4 Southern Research, Birmingham, AL, United States; 5 HudsonAlpha Institute for Biotechnology, Huntsville, AL, United States; 6 College of Nursing, University of South Alabama, Mobile, AL, United States; 7 Department of Medicine, The University of Mississippi Medical Center, Jackson, MS, United States; 8 Epidemiology Department, Tulane University, New Orleans, LA, United States; 9 School of Social Work, University of Alabama, Tuscaloosa, AL, United States; 10 Louisiana State University Pennington Biomedical Research Center, Baton Rouge, LA, United States; 11 Health Policy and Systems Management, LSU Health Sciences Center, School of Public Health, New Orleans, LA, United States; 12 Health Outcomes Research and Policy Department, Auburn University, Auburn, AL, United States

**Keywords:** capacity building, dissemination and implementation, mixed methods approach, stakeholder engagement, translational science

## Abstract

**Background:**

Dissemination and implementation (D&I) science is essential for translating evidence into practice, yet capacity remains uneven across Clinical and Translational Science Award (CTSA) hubs and networks. The University of Alabama at Birmingham Center for Clinical and Translational Science (CCTS) serves an 11-institution, multi-state Partner Network.

**Methods:**

We conducted a multi-phase assessment across the CCTS Partner Network. First, we performed qualitative interviews (April–June 2,025) with 18 stakeholders representing diverse roles and institutions to identify activities, barriers, and needs. Findings informed a needs assessment survey (February–March 2026) administered across the network (*N* = 202). Descriptive statistics summarized participant characteristics and priorities for training and consultation.

**Results:**

Qualitative findings revealed heterogeneous but growing D&I capacity, with gaps in training, infrastructure, and evaluation support. Participants reported limited formal training, strong demand for applied skills (e.g., study design, mixed methods, grant writing), and challenges in real-world implementation. Strong interest emerged in flexible, practice-oriented training and consultation services. Survey results reinforced these findings: although 49% of respondents identified as contributing to D&I, only 20% reported high expertise. Nearly half expressed strong interest in intensive short courses, particularly in frameworks, outcomes, and measurement. Consultation demand was high (77%), especially for study design, implementation strategies, and evaluation.

**Conclusions:**

There is strong engagement in D&I science, but gaps in expertise, infrastructure, and support persist. A coordinated strategy integrating structured training with centralized consultation and collaboration mechanisms is needed to strengthen D&I capacity. These findings informed implementation initiatives and illustrate a pragmatic approach that may inform D&I capacity-building efforts in other CTSA environments.

## Introduction

Dissemination and implementation (D&I) science has emerged as a critical component of translational research, aimed at accelerating the uptake of evidence-based interventions into routine practice and improving population health outcomes (1–3). Despite its growing importance, D&I capacity remains uneven across Clinical and Translational Science Award (CTSA) hubs and their affiliated networks, reflecting variability in infrastructure, workforce expertise, and access to training, consultation, and collaborative opportunities (4–7). CTSA programs are uniquely positioned to address these gaps by leveraging multi-institutional partnerships to support bidirectional engagement, identify context-specific needs, and build sustainable D&I capacity across diverse settings (8, 9).

With administrative leadership from a hub at the University of Alabama at Birmingham (UAB), the Center for Clinical and Translational Science (CCTS) serves a multi-state region encompassing Alabama, Mississippi, and Louisiana, and includes a Partner Network of 11 institutions spanning academic medical centers, public and private universities, research and biotechnology institutes, and healthcare systems. This geographic and organizational diversity introduces both opportunities and challenges for D&I capacity building, as partners vary substantially in their existing research infrastructure, experience with D&I science, and access to resources. Consequently, a one-size-fits-all approach is insufficient; instead, tailored strategies are required to systematically assess the current landscape and identify needs related to training, learning communities, methodological consultation, and cross-institutional collaboration. In response to growing demand, the CCTS established a D&I Partner Network Council, with representation from each participating institution. The Council began meeting monthly in October 2024 and serves as a central forum for coordination and engagement across the network. Its roles include facilitating information sharing on ongoing D&I activities, advising on the development and implementation of network-wide initiatives, and providing structured feedback to ensure that programs and resources are aligned with the needs and priorities of partner institutions. Developing an accurate understanding of these dimensions is essential to inform targeted investments and to ensure that D&I resources are responsive to the priorities of network partners.

To address this need, we implemented a multi-phase, stakeholder-engaged assessment within the CCTS Partner Network. First, the project team conducted a series of structured one-on-one “Meet and Greet” key informant interviews with Partner Network Council members to obtain qualitative insights into ongoing D&I activities, institutional strengths, and perceived barriers at their institutions. These discussions informed the development of a comprehensive needs assessment survey designed to systematically capture partners' priorities across key domains of D&I capacity, including training, technical assistance, consultation, and collaboration. By integrating qualitative and quantitative data, this effort describes a stakeholder-engaged approach for assessing D&I capacity and informing implementation planning within a CTSA Partner Network, with potential applicability to other academic research organizations.

## Methods

Because the objective was exploratory organizational assessment rather than evaluation of implementation determinants or implementation outcomes, the study was not organized around a single dissemination or implementation framework. Instead, a stakeholder-engaged mixed-methods approach was used to identify locally relevant priorities that will inform future implementation planning.

Because partner institutions varied substantially in size and research workforce, proportional representation across institutions was neither expected nor an objective of the organizational needs assessment.

### Qualitative phase: meet and greet key informant interviews

Between April and June 2025, 18 faculty and staff from 11 partner institutions participated. Participants were purposively sampled to ensure representation across all CCTS partner institutions and diverse stakeholder roles, including administrative leaders, principal investigators, early- and mid-career researchers, program developers, educators, and evaluation specialists. Larger partner institutions were represented by multiple participants to capture diverse perspectives and organizational functions. Individuals were identified through network leadership and existing professional connections to ensure representation of institutions with varying levels of D&I capacity.

Qualitative data were collected through one-on-one interviews using a semi-structured guide. The interview guide was developed by the study team based on the project objectives and implementation science capacity-building literature (10–12). The guide is provided in Supplementary Appendix A. Topics included definitions and perceptions of D&I, current research and practice activities, training experiences, infrastructure and support needs, and opportunities for collaboration. Discussions were conducted virtually through Zoom, documented through detailed notes, and supplemented with participant quotes to preserve context and meaning.

We used a rapid qualitative analysis approach appropriate for implementation planning and organizational needs assessments (13, 14). This approach was selected to generate actionable findings efficiently while maintaining a systematic analytic process. Two members of the study team reviewed interview notes and developed preliminary codes using an inductive approach. Coding discrepancies were resolved through discussion until consensus was reached. Codes were then organized into higher-order themes reflecting D&I capacity, training and education, support services, and collaboration (15, 16). Representative quotations were selected to illustrate key findings.

### Needs assessment survey

Building on themes identified during the key informant “Meet and Greet” interviews, we conducted a focused, cross-sectional needs assessment survey targeting two priority domains: (1) education and training needs and (2) demand for consultation services. This approach was designed to generate actionable, program-relevant data. Survey items were developed from themes emerging during the qualitative phase and reviewed by the Partner Network Council for content relevance prior to dissemination. The complete survey instrument is provided in Supplementary Appendix B.

The survey was administered over a six-week period (February 2–March 14, 2026) to faculty, researchers, trainees, and staff across the CCTS partner network. The survey was disseminated through the CCTS Partner Network using the CCTS newsletter and targeted outreach by Partner Network Council members. Because the survey link was distributed through multiple institutional communication channels and could be further shared within partner organizations, the total number of individuals who received the invitation could not be determined; therefore, a response rate was not calculated.

The instrument captured information across three domains: (1) participant characteristics, including institutional affiliation, professional role (e.g., faculty, staff, trainee), and primary research focus areas (e.g., health equity, behavioral health); (2) experience with dissemination and implementation (D&I) science, including self-identification as a D&I contributor and self-reported familiarity with core concepts; and (3) training and consultation needs, including interest in intensive short courses, foundational D&I topics, applied skill development (e.g., grant writing, study design), and preferred delivery formats (e.g., virtual, in-person, hybrid).

Consistent with the descriptive objectives of an organizational needs assessment, analyses focused on descriptive statistics to characterize stakeholder perspectives and identify areas of need and priority, rather than inferential hypothesis testing (17, 18). Frequencies and percentages were calculated for categorical variables to summarize participant characteristics and reported needs. For items assessing training and consultation priorities, top box or top 2 box score across a 5-reponse Likert scale was used to identify high-priority areas, defined as the proportion of respondents selecting “Very Interested/Somewhat Interested” or “Very Valuable/Somewhat Valuable.” Open-ended “Other” responses were reviewed and coded to ensure comprehensive representation of research focus areas and professional roles.

## Results

### Qualitative phase (meet and greet interviews)

#### Participant characteristics and institutional context

Participants represented diverse roles spanning leadership, research, education, and program implementation. Institutions varied widely in D&I capacity, ranging from nascent efforts with minimal infrastructure to well-established programs with formal implementation cores, training grants, and mentorship structures.

Theme 1: Heterogeneous but Growing D&I Capacity

Leadership and Infrastructure Development. Many institutions reported active efforts to expand D&I capacity, including hiring faculty with D&I expertise, establishing D&I cores, and building partnerships with clinical and community stakeholders. However, investment levels were uneven. Some institutions described strong leadership and strategic direction, whereas others characterized their efforts as fragmented and lacking sustained support.

“We're at a place where there's growing interest, but we need a longer-term strategy to build implementation science capacity. Right now, it's piecemeal.”

Breadth of D&I Activity. Participants reported D&I-related work across diverse domains, including chronic disease, maternal and child health, behavioral health, health equity, global health, and precision medicine. While some investigators led dedicated D&I initiatives (e.g., pragmatic trials, hybrid designs, training grants), others embedded D&I approaches within broader research programs without formally identifying as D&I researchers.

“Much of what we do in global health is implementation work—we just didn't always call it that.”

Implementation Practice Challenges. Common challenges included integrating interventions into clinical workflows, addressing de-implementation, and measuring fidelity and adaptation. Participants also highlighted gaps in evaluation capacity and tools, particularly in real-world settings. Contextual challenges were pronounced in community and global health settings, where resource constraints, system variability, and the need for local capacity-building influenced implementation processes.

“We're often still focused on bringing new programs in, but there's little guidance on when to scale back or stop what isn't working. That's a real need.”

“We have to bring people in from the outside to deliver programs in underserved areas. We need to build local capacity and long-term trust.”

Theme 2: Substantial Gaps in D&I Education and Training

Current Training Landscape. Training opportunities varied widely. Some institutions offered formal coursework, postdoctoral programs, or were developing certificate programs, while others relied on informal learning through project-based experience or mentorship. Many participants, particularly mid-career faculty, reported limited formal training in D&I.

“I've picked up bits and pieces from KL2 workshops and projects, but I wouldn't say I've been formally trained in Dissemination and Implementation Science.”

“I've had some exposure through collaborating on a project that used the CFIR framework, but no formal coursework. It's been mostly trial and error.”

Unmet Training Needs. Participants identified needs across multiple domains:

- Foundational knowledge (frameworks, study design, and methods)
- Applied skills (mixed methods, dissemination strategies, implementation trials)
- Context-specific training (e.g., global health, equity-focused approaches)

“I'm a mixed methods researcher, but I struggle with how to apply it specifically in implementation work. A workshop that walks through real use cases would be very helpful.”

“We need something that's not just for doctoral students. There are junior faculty who are already funded, but they need this foundation.”

Training needs differed by career stage. Early-career investigators sought structured mentorship and grant-writing guidance, while mid-career faculty and clinicians preferred concise, practice-oriented training formats.

Preferred Training Formats. There was strong preference for flexible and applied formats, including asynchronous modules, hybrid short courses, and intensive workshops. Participants emphasized the importance of practical learning experiences (e.g., proposal development, real-world case applications), along with access to toolkits, templates, and mentorship.

Theme 3: Gaps in Infrastructure and Support Services

Consultation Needs. Participants expressed high demand for expert consultation to support D&I project design and implementation. Key areas included framework selection, integration of D&I into existing programs, process evaluation, and clarification of implementation outcomes.

“It is such a broad and amorphous field. It would be incredibly helpful to identify which of community-based projects actually fit within Dissemination and Implementation frameworks.”

Grant Development and Peer Learning. Many investigators reported difficulty incorporating D&I aims into grant proposals, particularly for NIH mechanisms. Suggested supports included structured workshops, iterative feedback, and regular “drop-in” consultation sessions to facilitate proposal development and peer exchange.

Measurement and Evaluation Capacity. There was a consistent need for improved infrastructure to assess implementation outcomes such as fidelity, adaptation, and reach. Participants noted that existing evaluation resources were often insufficient for capturing the complexity of real-world implementation.

Sustainability and Policy Guidance. Participants emphasized the lack of tools and expertise for sustainability planning. Needs included guidance on long-term funding strategies, integration into health systems, and alignment with policy mechanisms.

“Can we learn from those who have figured out what policies or partnerships have actually helped sustain evidence-based programs?”

Theme 4: Strong Demand for Collaboration and Network Development

Communication and Visibility. Awareness of network resources and activities varied. Participants called for improved communication and clearer pathways for engagement to enhance participation across institutions and disciplines.

“I know we're supposed to be part of the Dissemination and Implementation Science network, but it's not always clear what that means or how we contribute.”

Cross-Sector Collaboration Opportunities. Participants highlighted several mechanisms to facilitate collaboration:

- Centralized directories of investigators, projects, and expertise
- Structured mentorship programs across career stages
- Regular networking forums and thematic workgroups
- Cross-institutional training and educational activities

Emerging Priorities. Key priorities included sustaining D&I programs amid funding constraints, tailoring D&I approaches to diverse contexts (particularly global settings), maintaining methodological innovation, and integrating new faculty into D&I networks early in their careers.

#### Needs assessment survey results

A total of 270 responses were received, of which 202 were complete and included in the analysis. Respondents represented 9 of the eleven CCTS partner institutions, with the largest proportions from the University of Alabama at Birmingham (28%), Tulane University (23%), the University of South Alabama (12%), and the University of Alabama (Tuscaloosa) (10%). Representatives from the remaining two partner organizations, a local technology institute and a biotechnology/research organization, participated in the qualitative interview phase but did not complete the survey. The sample was predominantly composed of faculty (61%), followed by research staff (13%), graduate students (10%), and administrators (9%). The most frequently reported research focus areas were health equity and social determinants of health (38%), behavioral health (36%), and maternal and child health (23%) (Table 1).

**Table 1:** Characteristics of survey respondents (*N* = 202).

Questions	Count (n)	Percentage (%)
Institutional Affiliation		
University of Alabama at Birmingham	56	28%
Tulane	47	23%
University of South Alabama	25	12%
Pennington Biomedical Research Center	20	10%
University of Alabama Tuscaloosa	19	9%
Auburn University	18	9%
University of Mississippi Medical Center	7	3%
Louisiana State University	5	2%
Tuskegee	2	1%
Other: Vanderbilt	2	1%
Other: YLABS	1	0.5%
HudsonAlpha Institute	0	0%
Southern Research	0	0%
Primary Professional Role*		
Faculty (Assistant, Associate, Professor)	123	61%
Research staff (Coordinator, Project Manager)	26	13%
Graduate student (Master's, PhD)	21	10%
Administrator/Program Manager	19	9%
Postdoctoral researcher/fellow	13	6%
Clinical practitioner	8	4%
Other	6	3%
Primary Research Focus Areas*		
Health equity/social determinants of health	75	38%
Behavioral health	72	36%
Maternal and child health	46	23%
Cardiovascular disease	34	17%
Metabolic disease	32	16%
Cancer	24	12%
Global Health	24	12%
HIV/AIDS	23	12%
Other (aging, environmental health, drug discovery, policy, etc.)	86	43%

* Participants could select more than one option; percentages may exceed 100%.

Nearly half of respondents (49%) self-identified as contributing to dissemination and implementation (D&I) science; however, only 20% reported being “very familiar” or “expert” in the field. The most common level of familiarity was “moderately familiar” (43%), indicating a substantial gap between engagement and depth of expertise. Primary motivations for pursuing D&I training included improving the translation of research into practice (75%), strengthening methodological skills (55%), and enhancing existing research projects (52%) (Table 2).

**Table 2:** D&I science familiarity, identity, and motivations.

Questions	Count (n)	Percentage (%)
Considers self a contributor to D&I science (*n* = 200)		
Yes	97	49%
No	54	27%
Not sure	49	25%
Familiarity with D&I Science (*n* = 199)		
Not at all familiar	25	13%
Slightly familiar	51	26%
Moderately familiar	85	43%
Very familiar	31	16%
Expert	7	4%
Primary Motivations for Learning D&I* (*n* = 200)		
Improve translation of research into practice	150	75%
Strengthen methodological skills in D&I	110	55%
Enhance existing research projects with D&I principles	104	52%
General professional development	90	45%
Incorporate D&I into a new grant submission	84	42%
To develop or evaluate health programs	71	36%
To expand my career opportunities	71	36%
To serve as a liaison for my institution/connect CCTS D&I resources with my institution's needs	20	10%

* Participants could select more than one option; percentages may exceed 100%.

There was strong interest in structured training opportunities. Nearly half of respondents (48%) reported being “very interested” in an intensive D&I short course. Foundational topics rated as most valuable included implementation outcomes and measurement, as well as D&I theories, models, and frameworks. Preferred training formats emphasized accessibility and practicality: synchronous online sessions (e.g., Zoom) were most favored for short courses, with preferred durations of a full day (27%), half-day (21%), or 2–3 days (21%). In terms of skill-based content, the highest interest was in D&I-focused grant writing and mixed methods research in D&I (Table 3).

**Table 3:** Interest and preferences for a D&I science short course.

Questions	Count (n)	Percentage (%)
Short course interest (*n* = 197) “*Very Interested*”	95	48%
Topics in short course (*n* = 171) “*Very Valuable*”		
Key D&I outcomes and measures	134	78%
D&I theories, models and frameworks	119	70%
Introduction to D&I Science conceptions and definitions	118	69%
Differences between D&I research and efficacy/effectiveness research	111	65%
Engaging stakeholders in D&I research	109	64%
Ideal duration (*n* = 169)		
Half day	36	21%
1 full day	46	27%
2 to 3 full days	36	21%
4 to 5 full days	3	2%
Delivered in 1–2 weeks (non-consecutive days)	21	12%
Delivered over 2 weeks (non-consecutive days)	27	16%
Delivery format, top rank (*n* = 160)		
Zoom	65	41%
In-person	49	31%
Asynchronous online (self-paced)	24	15%
Hybrid asynchronous/synchronous	13	8%
Combination of didactic learning with a hands-on project	9	6%

Demand for consultation services was also high, with 77% of respondents indicating they were “very” or “somewhat” interested in accessing such support. The most requested areas for consultation included study design, implementation strategies, and measurement and evaluation. A majority of participants preferred virtual consultation formats (54%), and nearly half (46%) expressed a preference for one-on-one sessions (Table 4).

**Table 4:** Interest in skill-specific trainings and consultation services.

Questions	Count (n)	Percentage (%)
Skill-Specific Training Interests (*n* = 194) “*Very Interested*”		
Grant writing for D&I proposals	112	58%
Mixed methods research in D&I	107	55%
Sustainability of interventions	100	52%
D&I in specific health contexts (e.g., chronic disease, mental health, etc.)	100	52%
Adapting interventions for new contexts	98	51%
Scale-up and spread of D&I interventions	85	44%
Economic evaluation in D&I	70	36%
Consultation Service Need (*n* = 183) “*Very Interested*”	141	77%
Consultation Service Topic Interests (*n* = 183) “*Very Interested or Somewhat Interested*”		
Study design	161	88%
Implementation strategies	159	87%
Measurement/evaluation	150	82%
Selection/application of frameworks	147	80%
Grant writing/funding opportunities	146	80%
Community/stakeholder engagement	133	73%
Preferred Consultation Type (*n* = 185)		
Individual	86	46%
Group	32	17%
Both	67	36%
Preferred Consultation Format (*n* = 191)		
In-Person	20	10%
Virtual	104	54%
No Preference	67	35%

## Discussions

We report interview and survey findings from a 3-state, 11-site CTSA network to inform strategic initiatives and tactical programming to enhance regional capacity building to support the conduct of robust D&I science projects and collaborations. Findings from the Meet and Greet interviews and the needs assessment survey collectively indicate a network with strong interest and momentum in D&I science, yet substantial heterogeneity in capacity, infrastructure, and expertise. While some institutions have established robust D&I ecosystems, others remain in early stages of development, characterized by fragmented efforts and limited institutional investment. This variability underscores the need for coordinated, network-level strategies to ensure more equitable access to resources and support across the region.

A central and consistent finding across both data sources is the mismatch between growing demand for D&I and the availability of structured training and support. Although over half of survey respondents identified as contributors to D&I science, only a small proportion reported advanced familiarity or expertise. This perceived “expertise gap” suggests that many investigators are engaged in translational work without sufficient grounding in D&I methodologies. The qualitative findings further reinforce that training is often informal and inconsistent, particularly among mid-career faculty and clinician-researchers who are expected to incorporate D&I components into research without formal preparation. Addressing this gap will require scalable, flexible, and applied training models that accommodate diverse roles, career stages, and institutional contexts (19–21).

The strong interest in intensive short courses and foundational topics, particularly D&I frameworks, outcomes, and measurement, highlights the need to establish a structured educational infrastructure. Preferences for synchronous, virtual formats and shorter-duration sessions reflect the logistical realities of a geographically dispersed network and support the feasibility of centralized, cross-institutional training. Importantly, respondents also emphasized the value of applied, “grant-ready” skills, including study design and proposal development, indicating that training initiatives should prioritize practical application alongside conceptual knowledge. In response to this feedback, we launched an inaugural half-day Specific Aims Workshop in conjunction with the annual D&I Science Research Meeting in April 2026.

Beyond training, the findings point to critical gaps in infrastructure to support D&I practice. Participants consistently identified consultation services, evaluation support, and grant development assistance as essential components of a comprehensive D&I support system. The high level of interest in consultation, particularly for individualized, project-specific guidance, suggests that establishing a centralized consultation hub could play a pivotal role in enhancing both the quality and competitiveness of D&I-focused research. Such services may be especially impactful for investigators seeking to integrate D&I aims into existing projects or develop fundable proposals (6, 22).

Sustainability and policy alignment emerged as additional areas of need. Despite active implementation efforts, participants reported limited capacity to plan for long-term maintenance or to leverage policy mechanisms for scale. Integrating sustainability planning, health system alignment, and policy engagement into D&I support services represents a key opportunity to strengthen the real-world impact and durability of interventions (2).

The strong demand for collaboration further highlights the importance of network-level coordination. Both interview and survey findings point to the need for mechanisms that facilitate connections across institutions, disciplines, and career stages. Centralized directories, structured mentorship programs, and cross-institutional training initiatives could reduce silos and enhance knowledge exchange. Improving communication and visibility of available resources will be essential to maximize engagement and ensure equitable participation across the network (5, 23).

### Implications for practice and research

These findings suggest several priorities for strengthening D&I capacity within multi-institutional networks: (1) development of tiered, competency-based training programs that integrate foundational and applied content; (2) establishment of centralized consultation and evaluation support services; (3) expansion of grant development and mentorship opportunities; and (4) investment in platforms that facilitate collaboration and information sharing. Future research should assess the effectiveness of these strategies in improving D&I capacity, increasing grant success, and accelerating the translation of evidence into practice.

Although this work was conducted within a single CTSA Partner Network, the transferability lies less in the specific capacity-building priorities identified than in the systematic stakeholder-engaged process used to identify them. Differences in institutional structure, organizational maturity, available resources, and existing D&I capacity will likely influence local priorities across CTSA hubs. Therefore, rather than representing a prescriptive model, this work illustrates a pragmatic approach that other CTSAs may adapt and contextualize to guide their own D&I capacity-building efforts. Because capacity-building priorities are inherently context dependent, organizations adopting this approach should intentionally engage stakeholders representing diverse institutional types, professional roles, and levels of implementation science experience. Future evaluations across multiple CTSA settings will be important for understanding the broader applicability of this approach.

### Limitations

This study was intentionally conducted as an exploratory organizational needs assessment rather than a framework-based implementation study. Applying a predefined framework at this stage may have constrained identification of stakeholder priorities, therefore, an inductive mixed-methods approach was used to characterize local needs. The qualitative component was based on a purposive sample and may not capture the full range of perspectives across the network. Data were derived from detailed notes rather than verbatim transcripts, which may limit analytic depth. The survey component is subject to self-selection bias, as individuals with existing interest in D&I may have been more likely to participate. Survey participation was also uneven across partner institutions and professional roles, reflecting differences in institutional size, existing research infrastructure, and engagement with CCTS activities. While all eleven partner institutions were represented during the qualitative interview phase, only nine institutions were represented in the survey responses. Accordingly, the findings should be interpreted as informing organizational capacity-building priorities rather than representing the perspectives of all Partner Network members. Additionally, the six-week survey period may not have reached all potential stakeholders across partner institutions. Lastly, because the survey was disseminated through multiple institutional communication channels, the total number of individuals receiving the invitation was unknown, precluding calculation of a response rate. Nevertheless, more than 200 responses were received from participants representing multiple partner institutions, professional roles, and research areas, providing broad input to inform capacity-building priorities.

## Conclusion

The CCTS partner network demonstrates strong motivation to advance D&I science but requires more structured, accessible, and practice-oriented support. A coordinated strategy that combines virtual, synchronous training with individualized consultation services has the potential to substantially strengthen D&I capacity. With targeted investment in training, infrastructure, and collaboration, the network is well-positioned to accelerate the translation of evidence into sustained population health impact. The approach taken and findings elicited may inform other CTSA hubs or similar entities in efforts to establish or expand multi-site regional D&I Science capacity and consortia.

## Data Availability

The raw data supporting the conclusions of this article will be made available by the authors, without undue reservation.
