# Supplementary material for: Building capacity and advancing collaboration in dissemination and implementation science: insights from a multi-institutional partner network
Source: Front Health Serv. 2026 Sep 7;6:1877155. doi: 10.3389/frhs.2026.1877155 (PMC13590565; doi:10.3389/frhs.2026.1877155)
Supplement: Supplementary file 1 [file Table1.docx]

**Appendix 1. CCTS D&I Section Partners Network Meet and Greet Interview Questions**

Opening & Context

1. Can you tell us about your role and how your work connects to dissemination and implementation research?
2. What are some key implementation challenges or questions your organization or your team is currently working on, when it comes to putting research into practice?

Training & Capacity Building

3. What types of D&I training does your organization currently offer?
4. What kinds of D&I training or tools would be most useful to you and your team? Are there specific skills you’d like to build?
5. Have you participated in any D&I-related training before?
   a. If so, what worked well, and what could be improved?
6. What would make a D&I learning opportunity most valuable and actionable for you?

Implementation Support & Services

7. What kinds of D&I support services—such as consultation or toolkits—would help you most in your D&I work?
8. Are there any areas where you feel you could use more guidance?
9. How does your organization track whether implementation efforts are working? Are there tools or support that could make that easier?

Collaboration and Network Engagement

10. How do you think the CCTS D&I Section can best support cross-sector collaboration among researchers, practitioners, administrators, and policymakers?
11. Are there any emerging trends or pressing issues in implementation science that you think the CCTS D&I Section should focus on?
12. How could collaborative events (e.g., workgroups, learning sessions) be more effective?

Vision, Mission & Future Direction

13. What should be the CCTS D&I Council’s priorities?
14. If you could design one new initiative or service to boost D&I work across the network, what would it be?
15. Is there anything you would like to learn from other CTSA networks?

Closing Questions

16. Is there anything else you’d like to share about your work or how the network can better support your dissemination and implementation efforts?
17. Anything important we haven’t asked that you think we should consider?

**Appendix 2. CCTS Dissemination & Implementation (D&I) Science Needs Assessment Survey**

**Dear Colleague,**

Thank you for being a valued member of the CCTS Dissemination & Implementation (D&I) Science Partner Network Council!

Informed by the feedback received from our “meet and greet” interviews with Council members, we are conducting a brief needs assessment survey to better understand your **learning and training needs** within Dissemination and Implementation (D&I) Science. Your insights will help us shape future programming, resources, and collaborative opportunities to support your work.

If you know others in your network who have an interest in D&I science, we encourage you to forward this brief survey to them as well — your help in expanding our reach is greatly appreciated.

Thank you in advance for your time and input!

**Part 1: Your Background**

1. Which university or organization are you primarily affiliated with? (drop down)

Auburn University

HudsonAlpha Institute

Louisiana State University

Pennington Biomedical Research Center

Southern Research

Tulane University

Tuskegee University

University of Alabama (Tuscaloosa)

University of Alabama at Birmingham

University of Mississippi Medical Center

University of South Alabama

Other: specify __________________

2. What is your primary role at your university? (Select all that apply)
   - Faculty (e.g., Assistant Professor, Associate Professor, Professor)
   - Postdoctoral Researcher/Fellow
   - Graduate Student (Master's, PhD)
   - Clinical Practitioner (e.g., Physician, Nurse, Allied Health Professional)
   - Research Staff (e.g., Research Coordinator, Project Manager)
   - Administrator/Program Manager
   - Other (Please specify): _____________________________________
3. What is your rank (ASKED ONLY OF FACULTY)?
   - Assistant Professor
   - Associate Professor
   - Professor
4. Please indicate your focus area(s) within D&I Science. Select all that apply
   - Behavioral Health
   - Cancer
   - Cardiovascular disease
   - Global health
   - Health equity/social determinants of health
   - HIV/AIDS
   - Maternal and child health
   - Metabolic disease
   - Other (specify): _____________________________________
5. Many people conduct work that moves research findings into real-world settings, but not everyone identifies as an Implementation Scientist. Have you ever considered yourself to be a person who contributes to or does D&I science?

- Yes
- No
- Not sure

6. How familiar are you with D&I Science?
   - Not at all familiar
   - Slightly familiar (heard of it, but limited understanding)
   - Moderately familiar (understand key concepts, but limited application)
   - Very familiar (actively incorporate D&I in my work)
   - Expert (lead D&I research/practice)
7. What are your primary motivations for learning more about D&I Science? (Select all that apply)
   - To incorporate D&I into a new grant submission
   - To enhance existing research projects with D&I principles
   - To improve the translation of research findings into practice (e.g., clinical settings, policy, and programs)
   - To develop or evaluate health programs
   - To strengthen my methodological skills in D&I
   - To expand my career opportunities
   - General professional development
   - Other (Please specify): _____________________________________

**Part 2: Short Course**

8. Please indicate your interest in an intensive D&I Science short course. This intensive short course offers **foundational training** in Dissemination and Implementation (D&I) Science and is designed to be completed in just a few days. It provides a focused overview of core theories, concepts, frameworks, and methods used to translate research into practice.
   - Very interested
   - Somewhat interested
   - Not too interested
   - Not at all interested
   - Don’t know
9. If a short course were offered, how valuable would these foundational topics be for you? (Select all that apply)

Use scale: very valuable, somewhat valuable, not too valuable, not at all valuable, and don’t know for the topics below:

- Introduction to D&I Science concepts and definitions
- D&I theories, models, and frameworks
- Key D&I outcomes and measures
- Differences between D&I research and efficacy/effectiveness research
- Engaging stakeholders in D&I research
- Other (Please specify): _____________________________________

10. What is the ideal duration for a short course?
    - Half-day
    - 1 full day
    - 2-3 full days
    - 4-5 full days
    - 1-2 weeks (part-time)
    - Longer than 2 weeks (part-time)
11. What is your preferred delivery format for a short course on D&I Science?

(Select all that apply, ranking your top 3 choices from 1 to 3 if possible, with 1 being most preferred)

- ___ In-person for a short course
- ___ Zoom/online (synchronous, live sessions)
- ___ Asynchronous online (self-paced)
- ___ Hybrid asynchronous/synchronous (participants do readings/lectures on their own, with a few synchronous meetings)
- ___ Combination of didactic learning with a hands-on project (e.g., write a grant, develop a methodology, etc.)
- Other (Please specify): _____________________________________

**Part 3: Skill-Specific Trainings**

12. How interested are you in skill- and topic-specific learning in Dissemination and Implementation Science? These learning opportunities would provide **advanced learning and greater depth** **in specific topics** than the short course that provides a foundational overview of Dissemination and Implementation Science.

Please indicate your interest in obtaining skill-specific and/or topic-specific D&I trainings.

Use scale: very interested, somewhat interested, not too interested, not at all interested, and don’t know for the topics below:

- Study designs for D&I research (e.g., stepped wedge, hybrid effectiveness-implementation)
- Mixed methods research in D&I
- Economic evaluation in D&I
- Adapting interventions for new contexts
- Sustainability of interventions
- Scale-up and spread of D&I interventions
- D&I in specific health contexts (e.g., chronic disease, mental health, global health)
- Grant writing for D&I proposals
- Other (Please specify): _____________________________________

**Part 4: Consultation Services Unit**

The Consultation Service Unit offers expert guidance to those seeking to integrate D&I Science into their work. Consultants provide support across a range of activities, including grant and manuscript development, selection and application of D&I frameworks and methods, etc.

13. How interested are you in scheduling a consultation service if you need one in the future?
    - Very interested
    - Somewhat interested
    - Not too interested
    - Not at all interested
    - Don’t know
14. Please rate your interest level in the following types of consultation services.

Use scale: very interested, somewhat interested, not too interested, not at all interested, don’t know

- Grant writing/funding opportunities
- Study design
- Selection/application of frameworks
- Implementation strategies
- Measurement/evaluation
- Community/stakeholder engagement
- Other (specify)

15. Which type of consultation would you prefer?
    - Individual consultation
    - Group consultation
    - Both
16. What is your preferred format for consultation services?
    - In-person
    - Virtual
    - No preference
17. Please provide any additional comments or suggestions you have about D&I Science learning opportunities.

______________________________________________________________

______________________________________________________________

______________________________________________________________

Thank you for your time and valuable input!

Please share this survey with your colleagues who have an interest in Dissemination and Implementation science.
